# Linggui Qihua Decoction Inhibits Atrial Fibrosis by Regulating TGF-*β*1/Smad2/3 Signal Pathway

**DOI:** 10.1155/2023/3764316

**Published:** 2023-02-11

**Authors:** Shuang Xiong, Yujiao Shi, Jiangang Liu, Chunqiu Liu, Lin Yang, Chenguang Yang, Guoju Dong

**Affiliations:** ^1^Xiyuan Hospital, China Academy of Chinese Medical Sciences, Institute of Cardiovascular Diseases, National Center for Clinical Cardiovascular Disease of Traditional Chinese Medicine, Beijing 100091, China; ^2^Graduate School of China Academy of Chinese Medical Sciences, Beijing 100700, China

## Abstract

Myocardial fibrosis is a critical factor in the development of heart failure with preserved ejection fraction (HFpEF). Linggui Qihua decoction (LGQHD) is an experienced formula, which has been proven to be effective on HFpEF in clinical and in experiments. *Objective*. This study aimed to observe the effect of LGQHD on HFpEF and its underlying mechanism. *Methods*. Spontaneously hypertensive rats (SHR) were induced with high-glucose and high-fat to establish HFpEF models and were treated with LGQHD for 8 weeks. The heart structure was detected by echocardiography, and the histopathological changes of the myocardium were observed by hematoxylin-eosin (HE) and Masson staining. Reverse transcription PCR (RT-PCR) and western blot were used to detect mRNA and protein expression of the target gene in rat myocardium. *Results*. In this study, LGQHD improved cardiac morphology and atrial fibrosis in HfpEF rats, decreased tissue inhibitor of metalloproteinase-1 (TIMP-1) mRNA expression, up-regulated matrix metalloproteinase-9 (MMP-9) mRNA expression, and inhibited the expression of angiotensin II (Ang II), angiotensin II type 1 receptor (AT1), transforming growth factor *β*1 (TGF-*β*1), Smad2/3 mRNA, and protein in myocardial tissue of HFpEF rats. *Conclusion*. LGQHD can suppress atrial fibrosis in HFpEF by modulating the TGF-*β*1/Smad2/3 pathway.

## 1. Introduction

Heart failure with preserved ejection fraction (HFpEF) is a special heart failure that has clinical symptoms and signs, abnormal cardiac structure and function, and elevated natriuretic peptides, but with normal ejection fraction (left ventricular ejection fraction, LVEF ≥50%) [1]. It was reported the overall morbidity of HFpEF in the general population is 1.1–5.5% [2], accounting for approximately 50% of all patients with heart failure [3]. Its morbidity and hospitalization are increasing annually [4, 5] and are expected to soon surpass that of heart failure with reduced ejection fraction (HFrEF) [6]. The current treatment for HFpEF includes angiotensin-converting enzyme inhibitor (ACE-I)/angiotensin-receptor blocker (ARB), beta-blockers, mineralocorticoid receptor antagonists (MRAs), and sacubitril/valsartan or sodium-glucose cotransporter 2 (SGLT2) inhibitors [1]. These treatments can improve certain specific phenotypes and reduce hospitalization rates in HFpEF. However, none of the large randomized controlled trials (RCTs) conducted in HFpEF have achieved their primary endpoints [1]. The SGLT2 inhibitor trials were conducted in HF patients with LVEF >40% [7]. Therefore, it is vital to explore the pathogenesis of HFpEF and develop potential medical therapies with improved therapeutic efficacy.

Hypertension and left ventricular hypertrophy stimulate interstitial cardiac fibrosis, which has long been considered to be a cause of passive muscle stiffening and reduced chamber compliance in HFpEF [8, 9]. Myocardial fibrosis also results from diabetic heart disease via multiple signaling cascades and alterations in extracellular matrix proteins, such as the formation of insoluble advanced glycation end-products [10]. Transforming growth factor *β* (TGF-*β*)/Smads signaling pathway is most closely related to the formation of myocardial fibrosis. TGF-*β* is the most important fibrous growth factor in the process of myocardial fibrosis. It activates the Smad2/3 signaling pathway mainly through binding TGF-*β* receptors to facilitate the myocardial fibrosis process [11]. As a key mediator of myocardial fibrosis, angiotensin II (Ang II) can up-regulate the expression of TGF-*β*1 by binding with angiotensin type 1 receptor (AT1R), which induces myocardial cell hypertrophy and enhances its secretion of profibrotic growth factor [12]. On the other hand, left atrial (LA) enlargement is common in HFpEF and correlates with the severity and duration of left ventricular (LV) diastolic dysfunction. In the setting of hypertensive HFpEF, LA remodeling occurs early and exhibits maladaptive alterations in LA compliance and left atrioventricular coupling which compromises overall cardiac performance and may exacerbate increases in LA and pulmonary pressures [13]. Therefore, exploring the potential roles of the TGF-*β*/Smads signaling pathway in the pathogenesis of HFpEF may provide a new method for the treatment of HFpEF.

Linggui Qihua decoction (LGQHD) is a patented and effective decoction for the clinical treatment of HFpEF which consists of *Poria cocos Schw Wolf* (Poria coco, 茯苓), *Cinnamomum cassia Presl* (*Cassia* twig, 桂枝), *Atractylodes macrocephala Koidz* (Atractylodes macrocephala, 白术), *Paeonia lactiflora Pall*., and *P*. *veitchii Lynch* (Radices paeoniae rubra, 赤芍). The whole formula is effective in warming yang for resolving fluid retention and promoting blood circulation and diuresis. Our previous studies showed that LGQHD can improve cardiac diastolic function, inhibit atrial fibrosis, as well as reduce inflammatory factors, and improve glucose and lipid metabolism and endothelial function in HFpEF model rats [14]. However, the mechanism underneath it is unclear. To explore the possible mechanism of LGQHD-treated HFpEF, we observe cardiac structure by echocardiography, the pathological changes of cardiomyocytes by Masson staining, and the activation of TGF-*β*/Smads signaling pathway in myocardial tissue by real-time polymerase chain reaction (RT-PCR) and Western blot analysis. Moreover, the potential mechanism of LGQHD for HFpEF was elucidated for the first time.

## 2. Materials and Methods

### 2.1. Animals

Thirty males and thirty females specific-pathogen-free (SPF) grade spontaneously hypertensive rats (SHR) and five males and five females with the same genetic background SPF Wistar-Kyoto rats (WKY) (age, 14 Weeks; weight, 180–260 g) were provided by the Vital Laboratory Animal Technology Company, Beijing, China (experimental animal license number: SCXK (Beijing) 2016-0006). These rats were housed in the barrier-class animal room of Xiyuan hospital of the Chinese Academy of Chinese Medical Sciences in a controlled environment (constant room temperature 23 ± 2°C, humidity 60% ± 10%, and 12 h light-dark cycle). The rats were fed on time, and the experiment was conducted after 1 week of adaptation. High-fat and high-sugar feed (10% lard, 10% sucrose, 2.0% cholesterol, 0.5% bile salt, and 77.5% basal feed) was manufactured by Beijing Co-operative Feeds Ltd. (feed certificate: Beijing Feed Certificate (2014) 06054).

### 2.2. Drugs Preparation and Chemical Composition Analysis

LGQHD is composed of *Poria cocos Schw Wolf* (Poria coco, 茯苓) 20 g, *Cinnamomum cassia Presl* (*Cassia* twig, 桂枝) 15 g, *Atractylodes macrocephala Koidz* (Atractylodes macrocephala, 白术) 10 g, *Paeonia lactiflora Pall*., and *P*. *veitchii Lynch* (Radices paeoniae rubra, 赤芍) 10 g. They are, respectively, derived from the dried mycorrhizal nucleus of *Poria coco*, the dried shoots of *Cassia twig*, the dried rhizome of *Atractylodes macrocephala,* and the dried roots of *Radices paeoniae rubra*. All herbs are purchased by Hebei Bai Cao Kang Pharmaceutical Co., Ltd. The preparation process consists of the traditional process of water extraction and volatile oil of the tablets to make *β*-CD inclusions in a ratio of about 1 : 4, and the aqueous solution is concentrated into an infusion. It was prepared as an extract at a concentration of 2.28 g of raw drug/g, which was provided by the Department of Pharmaceutics at Xiyuan hospital, China Academy of Chinese Medicine. Sacubitril Valsartan Sodium Tablets (trade name: Entresto) were manufactured by Novartis Pharma Schweiz AG with the (approval number H20170344, Switzerland) with the specification of 50 mg/tablet.

Waters (USAXevo)'s G2-S QTOF, quadrupole time of flight high-resolution mass spectrometer, and ACQUITY UPLC H-Class, ultraperformance liquid chromatograph, were used. A guard column and an ACQUITY UPLC HSS T3 column (2.1 × 100 mm, 1.8 *μ*m) were included in the column's setup.

The Traditional Chinese Medicine Preparation Department at Xiyuan hospital, Chinese Academy of Traditional Chinese Medicine, provides LGQHD Infusion. Precisely 90 ml of LGQHD extraction is taken, 5.5 ml of the 70% methanol solution is added, kept at room temperature for 30 minutes, and then it is filtered through a 0.22 *μ*m filter membrane. The LGQHD extract test solution was then made and kept in the refrigerator at a temperature of −20°C. The proper amount of LGQHD extraction solution is taken, it is liquefied with 70% methanol, and the sample for analysis is injected. Chromatographic circumstances mobile phase: gradient elution of water (*A*)-acetonitrile (*B*) as described in Table 1. The column temperature was 40°C, the flow rate was 0.4 ml/min, and the injection volume was 2 *μ*l. The ESI source, positive and negative ion scan, and scan range *m*/*z*: 50–1200 Da were the mass spectrometry conditions.

Data were acquired using Masslynx V4.1 software, and the resulting data were processed using Progenesis QI software and calibrated and peak aligned by peak detection and calibration processing algorithms, respectively. Meanwhile, the chemical formulae of the possible target compounds were imported into the software, and the following main components of the LGQHD were identified by qualitative analysis of the components through accurate molecular weight comparison.

### 2.3. LGQHD Extract Preparation

According to the weight, the compatibility ratio of the four medicines of *Poria coco*, *Cassia twig*, *Atractylodes macrocephala,* and *Radices paeoniae rubra* in LGQHD is 4 : 3 : 2 : 2. The drug was decocted three times with water and filtered through the process each time, and the filtrate to be combined finally. The extract was then further filtered to remove solid impurities and finally condensed into granules. According to the table of equivalent dose ratios converted by body surface area between humans and animals [15], based on a standard adult body mass of 70 kg, the dose for rats = standard adult clinical dose mg/kg × 70 kg × 0.018/200. The LGQH-H group was administered 9.92 g/kg/d, which is 2 times the clinically equivalent dosage. The LGQH-L group was administered 4.96 g/kg/d, which is the clinical equivalent dosage.

### 2.4. Main Reagents and Instruments

Streptozotocin (STZ) is manufactured by Merck Millistore (Germany). TRNzol total RNA extraction reagent is manufactured by Tiangen Biochemical Technology (Beijing, China) Co., Ltd. PrimeScript™ RT reagent Kit with gDNA Eraser, SYBR® Premix Ex Taq™ II (Tli RNaseH Plus), ROX plus DL2,000 DNA Marker is manufactured by TaKaRa Bio Co., Ltd. of Japan. The primer synthesis was provided by Invitrogen Fisher Technology (China) Co., Ltd. Goat Antirabbit IgG (*H* + *L*) and Goat Antimouse IgG (*H* + *L*) by Tiandeyue (Beijing, China) Biotech Co., Ltd., and *β*-actin Mouse Mumab by Immunoway, Inc. (US). Rabbit polyclonal antibodies of Angiotensin II-1 Receptor (AT-1R) (Lot No. GR3188953-16), Transforming Factor-*β*1 (TGF-*β*1) (Lot No. GR3237963-8), Leukocyte Inhibitory Factor 2/3 (Smad2/3) (Lot No. GR3240249-3), *α*-SMA protein (Lot No. GR53219-3) and Matrix Metalloproteinase-9 (MMP-9) (Lot No. GR3280484-1) were manufactured by Abcam (UK), type I collagen (Lot No.bs0578), type III collagen (Lot No. bs33129) were manufactured by Beijing Biosynthesis Biotechnology CO., LTD. (China) and provided by Beijing Kezhongzhi Biological Technology Development CO, LTD. (China). The rabbit monoclonal antibody of Angiotensin II (Ang II) (Lot No. 82190377–2) was provided by GeneTex (USA); the rabbit monoclonal antibody of phosphorylated leukocyte inhibitory factor 2/3 (P-Smad2/3) (Lot No. 82180405–2) by GeneTex (USA) and provided by Beijing Xin Bosheng Biotechnology Co., Ltd. The mouse monoclonal antibody of matrix metalloproteinase inhibitor-1 (TIMP-1) (lot No. UB275560) by Novusbio (USA) Biologics, Inc supplied by Beijing Kezhongzhi Biotechnology Development Company. Tanon gel imaging system (model 1600), manufactured by Shanghai Tanon Technology Co., Ltd. and applied biosystems fluorescent quantitative PCR instrument (model ABI7500), manufactured by Life TechnologiesLife Technologies (USA), Inc.

### 2.5. Animal Model and Groups

Fifty 14-week-old SHR rats were given a high-fat diet after 16 weeks of continuous feeding, and after 2 weeks of a high-fat diet, STZ (25 mg/kg body weight) was injected intraperitoneally, and the high-fat diet was continued to induce the establishment of the HFpEF model in rats [16–18]. Surviving model rats were grouped and the survival rate was approximately 80%.

The rats were randomly divided into SHR model group (SHR group) and HFpEF model group (HFpEF group). The rats were split into the SHR model group, the HFpEF model group, the positive drug control group (Entresto group), the LGQHD high-dose group, and the LGQHD small-dose group at random (LGQH-L group). In which the same volume of pure water was gavaged to the SHR group and the HFpEF group. Shakubatra valsartan sodium tablets, 18 mg/kg body mass, were gavaged to the Entresto group; 8.10 g of the raw drug were given to the LGQH-H group; and 4.05 g was given to the LGQH-L group. Each group contained 10 animals, and the medications under test were diluted and given in equal amounts for 8 weeks. In addition, the normal control group (normal group) of 10 WKY rats with the same genetic background was administered the same amount of pure water. Except for the SHR and WKY groups, the rats were fed with a normal diet, while the other groups continued to be fed with a high-fat and high-sugar diet. Four rats were sacrificed during the experiment.

The animal model and experiment processes were performed strictly in accordance with the recommendations in the Guide for the Care and Use of Laboratory Animals of the National Institutes of Health. This experiment was approved by the Committee on Ethics of Animal Experiments of Xiyuan Hospital of China Academy of Chinese Medical Sciences (No. 2018XLC004-2).

### 2.6. Echocardiography Parameters Measurements and Cardiac Histopathology

After the treatment, the rats were anesthetized intraperitoneally with 2% sodium pentobarbital aqueous solution (0.2 mL/100 g body weight) for 12 h on the 2nd day of an empty stomach, and after anesthesia, the rats were evaluated by echocardiography with a high-frequency line array ultrasound diagnostic instrument (GE VividE95 cardiac ultrasound diagnostic instrument, manufactured by General Electric Company, USA) for cardiac function and echocardiographic morphological changes. Cardiac ultrasound morphological indices include left atrium diameter index (LADI), such as the left atrium diameter/Left (DI/*L*) and the left atrium diameter/Right(DI/*R*), left atrium volume index (LAVI); right atrium diameter index (RADI), such as, namely, right atrium diameter/Left (DI/*L*), right atrium inner diameter/Right (DI/*R*), interventricular septal thickness (IVST), and left atrial ejection fraction (LAEF). The operation of ultrasound examination was completed by two experienced ultrasound specialists who were blind to the experiment.

Myocardial tissues of 5 rats were randomly selected from each group and fixed in 10% central formaldehyde solution, embedded in immersion wax, dewaxed in sections, debenzene in ethanol step by step, and then stained sequentially with hematoxylin-eosin (HE) stain/Masson stain, acid alcohol separation, 1% eosin stain, and finally debenzened in ethanol step by step and sealed in neutral gum. The cardiac structure was photographed by a gross sample imaging system (Beijing Ruizhiaoheng Vision Technology Co., LTD.), and the morphology of myocardial tissue was observed under an optical microscope (Olympus (Japan) Co., LTD.).

### 2.7. Quantitative Real-Time Reverse Transcription PCR (RT-PCR)

We use TRNzol total RNA extraction reagent for sample RNA extraction, then use the PrimeScript™ RT reagent Kit with gDNA Eraser was used for cDNA reverse transcription, and then RT-PCR samples were detected. The 96-PCR plate was placed on the RT-PCR instrument for PCR reaction, and the melting curve of PCR products was established. The experimental operation was carried out according to the product instructions. RT-PCR was used to detect 3 Wells in each sample, and the data were analyzed by 2^−ΔΔCT^ method. Primer sequences are shown in Table 1.

### 2.8. Western Blot Analysis

Precooled RIPA lysate was used for protein extraction, and a protease inhibitor was added along with a phosphatase inhibitor. The bicinchoninic acid method (Bicinchoninic acid, BCA) was used for protein concentration detection, along with protein concentration conversion and protein concentration adjustment, and electrophoretic stopping time was determined by prestaining protein marker. The primary antibody was incubated with 3% BSA-TBST dilution, incubated for 10 minutes at room temperature, and placed at 4°C overnight. TBST washes the film. ECL is added to the film and then the reaction film is exposed, developed, and fixed. The membrane was washed with stripping buffer, the internal reference was incubated, beta-actin murine monoclonal antibody was, respectively, added and the antibodies were diluted with 3% BSA-TBST, 1 : 5000 dilution for AT1 and AngII, 1 : 4000 dilution for TGF-*β*1, Smads2/3 and P-Smada2/3, 1.1 dilution for MMP-9 and TIMP-1: 1000, 1 : 4000 dilution for type Icollage, 1 : 2000 dilution for type III collagen, and 1 : 2000 dilution for *α*-SMA protein and incubated at room temperature. The membrane was washed again, incubated with secondary antibody, goat antimouse IgG (*H* + *L*) HRP, diluted antibody with 3% BSA-TBST at a ratio of 1 : 10000, shaken gently at room temperature for 40 minutes, and the membrane was washed. ECL was added to the membrane and reacted, the film was exposed for development and fixation, and a specific positive band could be detected. The gray value of Western blot protein expression was transformed from JPEG to TIF by the software image *J*. The integrated optical density (IOD) values of the reading strips were calculated using Total Lab Quant V11.5, manufactured in Newcastle upon Tyne, UK.

### 2.9. Statistical Analysis

SPSS 13.0 statistical software was used for statistical analysis. All data were expressed as the mean ± standard deviation (mean ± SD). One-way ANOVA was used to compare the measured data for each group when the data met the normal distribution and homogeneity of variance, and the LSD test was used for further multiple comparisons between groups. If the homogeneity of variance was not satisfied, the F value was corrected by the Welch method, and the Dunnett *T*3 method was used for further multiple comparisons. The difference was considered statistically significant at *P* < 0.05.

## 3. Results

### 3.1. Chemical Composition Analysis of the Main Constitutes in LGQHD Extracts

**Figure 1:**
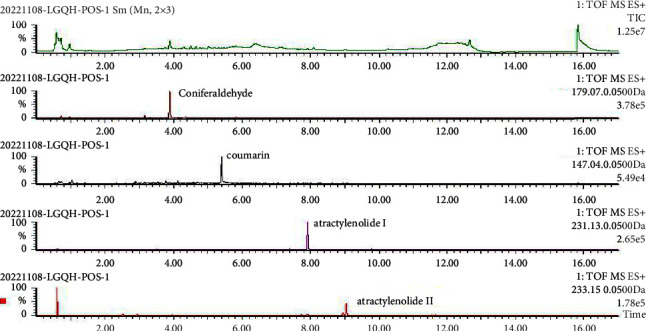
Base peak chromatogram (BPI) and extracted ion chromatogram (EIC/XIC) in positive ionization mode of LGQHD.

**Figure 2:**
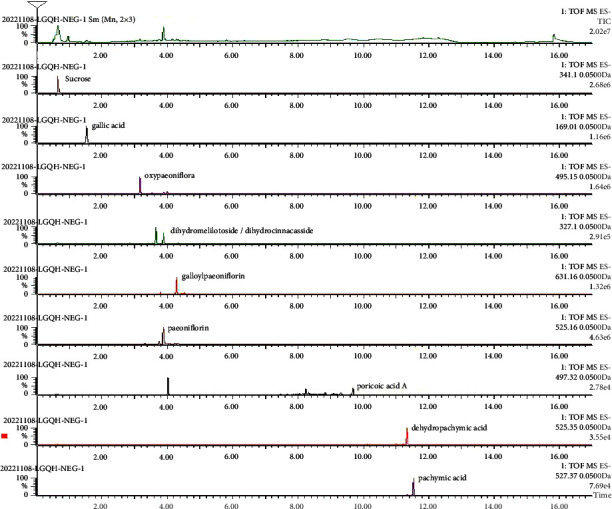
Base peak chromatogram (BPI) and extracted ion chromatogram (EIC/XIC) in negative ionization mode of LGQHD.

The LC-MS method was adopted to compare the compounds between the standard and LGQHD using positive and negative ion detection modes. The ion chromatograms of the main compounds and LGQHD are shown in Figures 1 and 2. The main compounds detected in positive ion mode were conifer aldehyde, coumarin, atractylenolide I, and atractylenolide II; the main compounds detected in negative ion mode were sucrose, gallic acid, oxypaeoniflora, dihydromelilotoside/dihydrocinnacasside, galloylpaeoniflorin, paeoniflorin, poricoic acid *A*, dehydropachymic acid, and pachymic acid.

### 3.2. LGQHD Improved Cardiac Ultrasound Morphology in HFpEF Rats

**Figure 3:**
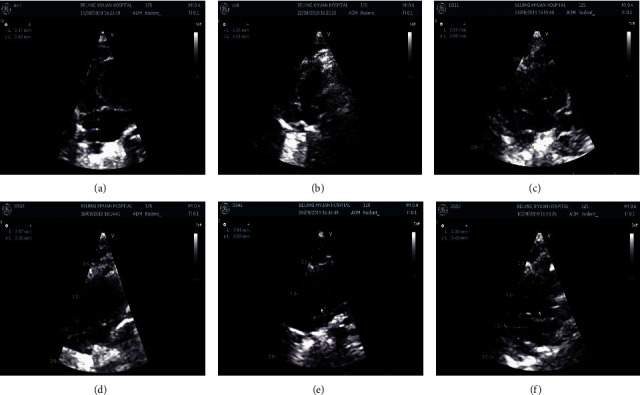
Effect of LGQHD on cardiac ultrasound morphology of HFpEF model rats. (a) WKY. (b) SHR. (c) HFpEF. (d) Entresto. (e) LGQH-H. (f) LGQH-L.

Echocardiography is an important means to detect the morphological changes of rat heart, and the results showed that compared with the control group (WKY group), the LAVI, IVST, LADI/R, and RADI/L of the model group (HFpEF group) were increased (*P* < 0.01, *P* < 0.05), indicating that the rat heart morphology changed after modeling. LAVI was decreased in the small and large dose groups of LGQHD and Entresto group (*P* < 0.05); LADI/R and RADI/R were decreased in the high-dose group of LGQHD (LGQH-H) (*P* < 0.05, *P* < 0.01), and LADI/R was decreased in the low-dose group of LGQHD (LGQH-L) (*P* < 0.01); the results showed that LGQHD improved cardiac morphological changes. The results are shown in Figure 3 and Table 2.

### 3.3. LGQHD Ameliorated Atrial Fibrosis in HFpEF Rat Models

**Figure 4:**
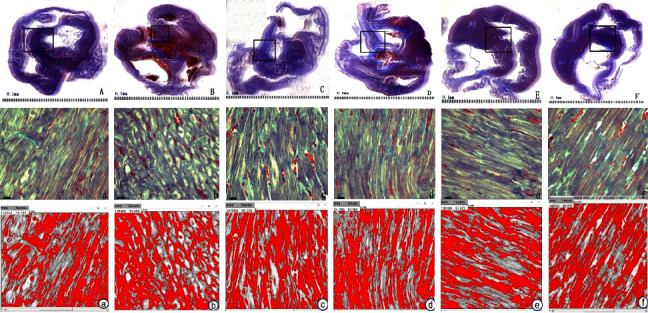
The effect of LGQHD on the morphology of atrial in rats (Masson, ×200). (Gross pathology: (A) WKY; (B) SHR; (C) HFpEF; (D) entresto; (E) LGQH-H; (F) LGQH-L; optical microscope and quantitative analysis: (a) WKY; (b) SHR; (c) HFpEF; (d) entresto; (e) LGQH-H; (f) LGQH-L;).

To verify the protective effect of LGQHD in the process of HFpEF atrial fibrosis, a high-glucose and high-fat induced HFpEF model in SHR rats was established, and the pathological changes of the myocardium were observed by Masson staining. This staining method detects the extent of collagen fibers deposition, and because it employs the combination of three dyes, can also distinguish muscle fibers (red), from collagen (blue) and nuclei (black), simultaneously. This method is suitable for the assessment of the extent and distribution of fibrosis in a quantifiable manner and can be successfully applied for the detection/diagnosis of fibrotic diseases [19]. The medical image analysis software Image *J* was applied to analyze the collagen fiber expression of each group and calculate the collagen volume fraction (CVF = collagen area/total area). The myocardial fibers of WKY rats were neatly arranged with normal cell morphology and a slightly proliferation of collagen fibers. In SHR rats, myocardial interstitial fibers were deposited, the myocardial cell arrangement gap was increased, and some collagen fibers were proliferated. In the model group, myocardial cells were arranged in a disorderly manner, and some myocardial cells were replaced by collagen fibers, collagen fibers were significantly increased, and the cell tissue gap was enlarged. After treatment in the Entresto group, LGQH-H group, and LGQH-L group, myocardial fiber collagen deposition was reduced and collagen fiber proliferation was decreased, especially in the LGQH-H group. The Image *J* results showed that compared with the control group, the myocardial tissue collagen area was larger and CVF was greater in the model group; after drug treatment, the myocardial tissue collagen fibers were reduced to different degrees in all groups. The results are shown in Figure 4.

### 3.4. LGQHD Regulates the Expression of Myocardial Fibrosis-Related Genes in Atrial Tissue of HFpEF Rats

**Figure 5:**
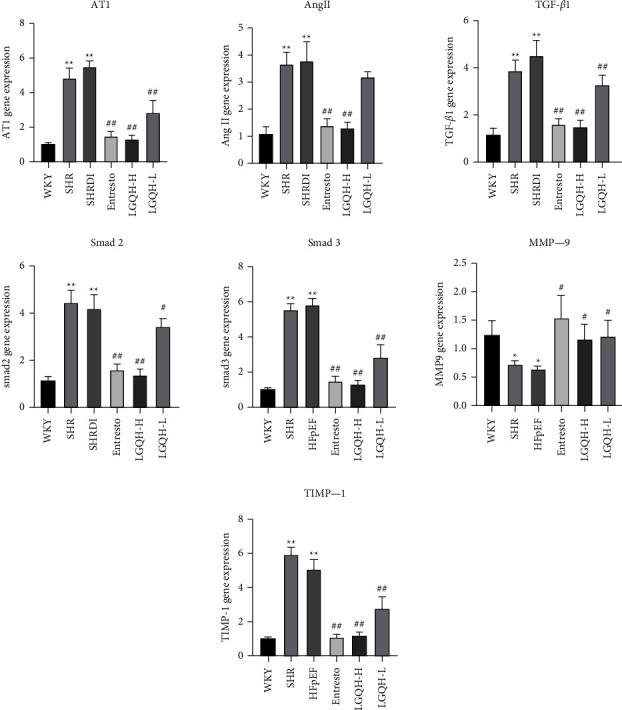
Effect of LGQHD on gene expression in myocardial tissue of HFpEF rats. (^*∗*^: Compared with WKY group, HFpEF *P* < 0.05. ^*∗∗*^: Compared with WKY group, HFpEF *P* < 0.01. ^#^: Compared with HFpEF group, each drug treatment group *P* < 0.05. ^##^: Compared with HFpEF group, each drug treatment group *P* < 0.01. AT1: angiotensin II type 1 receptor. AngII: angiotensin II. TGF-*β*1: transforming growth factor-*β*. MMP-9: matrix metalloproteinases. TIMP-1: tissue inhibitors of matrix metalloproteinase).

TGF-*β*/Smads signaling pathway plays an important role in the development of myocardial fibrosis. We detected AngII, AT1, TGF-*β*1, Smad2, Smad3, MMP-9, and TIMP-1 mRNA in the atrial tissue of HFpEF rats by RT-PCR level. Compared with the control group, the expression of MMP-9 mRNA was down-regulated (*P* < 0.05), while AngII, AT1, TGF-*β*1, Smad2, Smad3, and TIMP-1 mRNA expression were up-regulated in model group (*P* < 0.01). After drug treatment in each administration group, the expression of AT1, TGF-*β*1, TIMP-1, Smad2, and Smad3 mRNA were down-regulated in the LGQH-H group, LGQH-L group and Entresto group (*P* < 0.01), while the mRNA expression of MMP-9 was up-regulated (*P* < 0.05). The LGQH-H group and Entresto group could also down-regulate the expression level of AngII mRNA (*P* < 0.01), while the LGQH-L group had no obvious improvement on AngII mRNA. As shown in Figure 5.

### 3.5. LGQHD Regulates the Expression of Myocardial Fibrosis-Related Proteins in Atrial Tissue of HFpEF Rats

**Figure 6:**
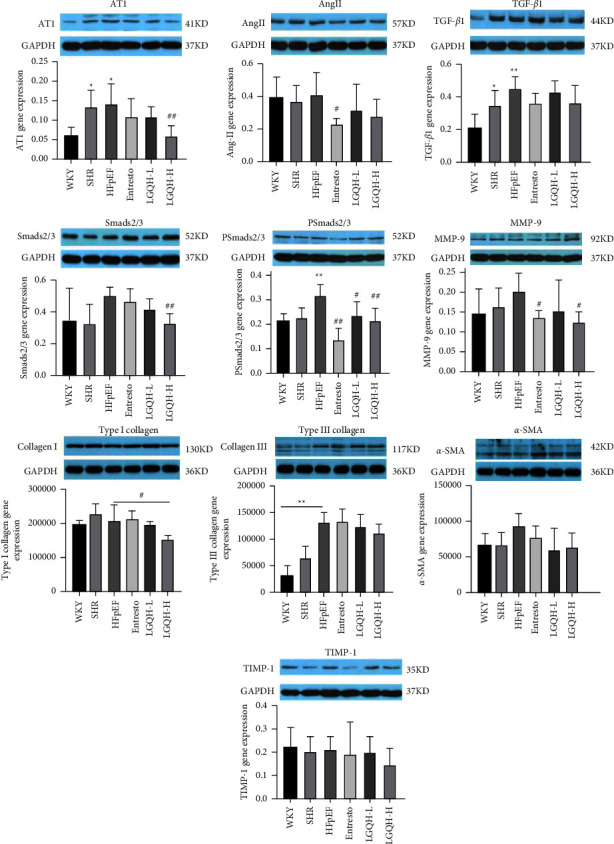
Effect of LGQHD on protein expression in myocardial tissue of HFpEF rats. (^*∗*^: Compared with WKY group, HFpEF *P* < 0.05. ^*∗∗*^: Compared with WKY group, HFpEF *P* < 0.01. ^#^: Compared with HFpEF group, each drug treatment group *P* < 0.05. ^##^: Compared with HFpEF group, each drug treatment group *P* < 0.01. AT1: angiotensin II type 1 receptor. AngII: angiotensin II. TGF-*β*1: transforming growth factor-*β*. MMP-9: matrix metalloproteinases. TIMP-1: tissue inhibitors of matrix metalloproteinase).

Western blot analysis the expression levels of AngII, AT1, TGF-*β*1, Smad2, Smad3, MMP-9, TIMP-1 proteins, Type I collagen, Type III collagen, and *α*-SMA protein in TGF-*β*/Smads signaling pathway in atrial tissue of HFpEF rats. Compared with the control group, the expression of TGF-*β*1, PSmad2/3, AT1 receptor, and Type III collagen protein were up-regulated in the model group (*P* < 0.01 or *P* < 0.05). Type I collagen and *α*-SMA protein expression were differentially upregulated. After 8 weeks of drug treatment, the LGQH-H group and Entresto group could down-regulate MMP-9 and PSmad2/3 protein expression (*P* < 0.05, *P* < 0.01), AT1 receptor and Smad2/3 protein expression were down-regulated in LGQH-H group (*P* < 0.01). It also down-regulated Type I collagen protein expression (*P* < 0.05). The expression of PSmad2/3 protein was down-regulated in the LGQH-L group (*P* < 0.05). LGQH-H and LGQH-L groups also downregulated Ang II, TGF-*β*1, TIMP-1, Type III collagen, and *α*-SMA protein expression, but the difference was not statistically significant. As shown in Figure 6.

## 4. Discussion

Myocardial fibrosis is a critical factor in the development of HFpEF, which refers to the proliferation of fibroblasts, excessive collagen synthesis, and deposition of extracellular matrix (ECM) in the structure of myocardial tissue [20]. In the myocardium, the extracellular matrix consists primarily of fibrillar collagen, and cardiac fibroblasts regulate the balance of extracellular matrix synthesis and degradation. Myocardial injury induces the differentiation of cardiac fibroblasts into myofibroblasts, and the expression of *α*-SMA indicates successful transformation into a phenotype that has a strong ability to synthesize extracellular matrix proteins [21]. Cardiac myofibroblasts contribute to the structural and functional changes in the heart by increased deposition of ECM components, predominantly collagen types I and III, within the interstitium by regulating autocrine/paracrine factors [22]. It was shown that decreased myocardial compliance and increased stiffness caused by myocardial fibrosis may be a potential mechanisms for diastolic dysfunction in HFpEF [23–26].

The expression balance between matrix metalloproteinases (MMPs) and tissue inhibitors of matrix metalloproteinase (TIMPs) plays a critical role in the maintenance of extracellular matrix [27]. MMP-9 and TIMP-1 are the most abundant MMPs and TIMPs in the myocardium, respectively. MMP-9 disrupts the collagen network architecture mainly by degrading type I and IV collagen and increasing type III collagen in the extracellular matrix, which causes the atrial wall to thin and then the atria to enlarge under pressure. The level of MMP-9 is positively correlated with the severity of myocardial fibrosis [28]. TIMP-1 can specifically inhibit the activity of MMP-9 and alleviate the degradation of extracellular matrix collagen, thus improving myocardial fibrosis. Under physiological conditions, MMP-9 and TIMP-1 maintain a balance at a ratio of 1 : 1. The balance is disturbed in pathological conditions, resulting in increased or decreased collagen degradation [29]. Thus, it accelerates or slows down the deposition of collagen fibers and aggravates or ameliorates atrial fibrosis.

In this study, the expression of MMP-9 mRNA was significantly down-regulated while the expression of TIMP-1 mRNA was significantly up-regulated in the myocardium of SHR rats after continuous high-sugar and high-fat diet and intraperitoneal injection of STZ for modeling. Masson staining showed that myocardial cells were disorganized, cellular tissue gap was increased. And, some of the myocardial cells were replaced by collagen fibers, so the collagen fibers were significantly increased. Furthermore, we observed significant upregulation of *α*-SMA protein and types I and III collagen expression, suggesting that cardiac fibroblasts had differentiated into myofibroblasts, resulting in increased collagen synthesis. It suggests that myocardial fibrosis occurred in the atrial tissue of rats after modeling. After 8 weeks of administration, the TIMP-1 mRNA and type I collagen expression were markedly down-regulated, both *α*-SMA protein and type III collagen expression were downregulated to different degrees, while the MMP-9 mRNA expression was significantly up-regulated in cardiac tissue of HFpEF rats. The decrease of collagen deposition and collagen fiber proliferation in myocardial fibers of rats under Masson staining indicated that LGQHD could effectively improve myocardial fibrosis. The protein levels of MMP-9 and TIMP-1 were inconsistent with the mRNA expression levels, which may be due to the fact that mRNA was easily degraded, exists in tissues for a short time and had stable properties after being translated into protein. Or, the regulation of MMP-9 and TIMP-1 were regulated by gene polymorphisms, and further analysis of susceptibility genes and susceptibility biomarkers was needed in the future.

Myocardial fibrosis is a complex pathological process involving multiple cytokine and molecular pathways, in which multiple pathways lead to different tissue molecular patterns of fibrosis, which in turn translate into diverse clinical phenotypes [30]. The major pathways are TGF-*β*/Smads, p38 MAPK, Wnt/*β*-Catenin, *G*protein-coupled receptor kinase (GRK), and Hippo [31], among which the TGF-*β*/Smads signaling pathway is most closely associated with myocardial fibrosis formation and is the key to the prevention and treatment of myocardial fibrosis [32]. TGF-*β* is part of the superfamily of growth factors. TGF-*β*1 is a key regulator in myocardial fibrosis, which can affect cell growth, apoptosis, and differentiation, increase ECM production, inhibit the production of MMPs, and reduce collagen degradation [33]. Smad proteins are TGF-*β* downstream signal molecule that mediates the signal transduction of TGF-*β*. At present, the TGF-*β*1/Smads signaling pathway is the most significant pathway in the process of myocardial fibrosis. TGF-*β*1 exacerbates myocardial fibrosis by binding to receptors, activating Smad2 and 3, and further translocating to the nucleus, up-regulating the expression of genes associated with ECM synthesis, and promoting ECM deposition [34]. Ang II is a well-known fibrogenic factor and has been associated with fibrosis in various organs, including myocardial fibrosis. The combination of Ang II and AT1 can induce TGF-*β*1 expression, cause cardiomyocyte hypertrophy and fibroblast proliferation, and consequently affect atrial fibrosis [35]. Conversely, it can also improve myocardial fibrosis by inhibiting the expression of TGF-*β*1 [36].

In this study, we found that the expression of AT1, TGF-*β*1, and Smad2/3 in myocardial tissues of rats in the small and large dose groups of LGQHD was significantly reduced, suggesting that the improvement of myocardial fibrosis by LGQHD may be related to the inhibition of TGF-*β*1/Smad2/3 signaling pathway. This is manifested that LGQHD can reduce the expression of Ang II and AT1 receptors, down-regulate the expression of TGF-*β*1 in cardiomyocytes, inhibit Smad2/3 expression, reduce Smad2/3 hyperphosphorylation, and negatively regulate the biological effect of TGF-*β*1 on myocardial fibrosis, and thus ameliorating myocardial fibrosis.

LGQHD is composed of four components *Poria coco*, *Cassia twig*, *Atractylodes macrocephala*, and *Radices paeoniae rubra*. *Poria coco* as the JUN, and the effect is to invigorate the spleen for diuresis, eliminating dampness and resolving fluid-retention;*Cassia twig* is the CHEN, the effect is to warm Yang and transforming Qi. The combination of *Poria coco* and *Cassia twig* is a common combination to promote Yang to transform Qi and diuresis, while *Atractylodes macrocephala* and *Radices paeoniae rubra* as ZUO and SHI. The whole formula builds up the effect of warming yang for resolving fluid-retention and promoting blood circulation and diuresis. Pharmacological studies have shown that *Poria coco* can improve hemodynamics and reduce cardiac preload in rats with myocardial hypertrophy and improve cardiac output and myocardial contractile function, as well as reduce left ventricular end-diastolic pressure, thus improving myocardial diastolic function, which can inhibit the development of pathological myocardial hypertrophy and delay the progression of heart failure [37]. *Cassia twig* can promote yang to transform qi, eliminate blood stasis and dissolve symptoms, and has a strong effect of diuresis and dehumidification. It can also increase urine output in mice, and the diuretic effect is longer than furosemide [38]. The combination of *Poria coco* and *Cassia twig* has been studied to inhibit myocardial hypertrophy and ventricular remodeling, improve myocardial fibrosis, as well as control the inflammatory response and inhibit neuroendocrine hyperactivation in rats with heart failure [39]. *Radices paeoniae rubra* has been shown to intervene in early ventricular remodeling after acute myocardial infarction, inhibit serum inflammation levels, protect ischemic cardiomyocytes, and promote recovery of cardiac function [40]. Its improvement of ventricular remodeling may be related to its ability to inhibit the TGF-*β*/Smad signaling pathway to reduce fibrosis [41–44]. *Atractylodes macrocephala* has the effects of immune regulation, anti-inflammatory, gastrointestinal mucosa protection, and blood sugar reduction [45]. Previous studies have found that the combination of Linggui Zhugan Decoction with the function of promoting blood circulation and removing stasis can significantly improve the clinical symptoms and cardiac function of HFpEF patients [46]. This was further confirmed in the research group's previous study on the effect of LGQHD on HFpEF rat models [14].

## 5. Conclusions

In this study, we identified the protective role of LGQHD in HFpEF. LGQHD can inhibit atrial fibrosis by suppressing the activation of TGF-*β*1/Smad2/3 signaling in HFpEF rats. Our findings provide a solid foundation for future investigation of the role of atrial fibrosis in HFpEF progression and the traditional Chinese medicine LGQHD is a promising candidate for the development of novel HFpEF treatments.

## Figures and Tables

**Table 1:** The gene primer sequence.

Primer name	Primer sequence (5′ to 3′)	Product size (bp)
AT1 receptor upstream primer	CAACTGCCTGAACCCTCTGT	129
AT1 receptor downstream primer	AGCGTGCTCATTTTCGTAGAC	

TGF-*β*1 upstream primer	GAGAGCCCTGGATACCAACTACTGC	93
TGF-*β*1 downstream primer	CAACCCAGGTCCTTCCTAAAGTCAA	

Smad2 upstream primer	TTTGCCGAGTGCCTAAGTGATA	211
Smad2 downstream primer	TTCTTATGGTGCACATTCGAGTC	

Smad3 upstream primer	GCTGTCTACCAGTTGACTCGCAT	135
Smad3 downstream primer	GGGTGCTGGTCACTGTCTGTCT	

MMP9 upstream primer	GGAACTCACACAACGTCTTTCACTA	236
MMP9 downstream primer	CAGGAGGTCATAGGTCACGTAGG	

TIMP-1 upstream primer	TTCCTGGTTCCCTGGCATAAT	295
TIMP-1 downstream primer	ATCGCTCTGGTAGCCCTTCTC	

AngIIupstream primer	CACCCCTTTCATCTCCTCTACTA	261
AngIIdownstream primer	TCTTGCCTCACTCAGCATCTT	

GAPDH upstream primer	TGGAGTCTACTGGCGTCTT	138
GAPDH downstream primer	TGTCATATTTCTCGTGGTTCA	

**Table 2:** Effect of LGQHD on cardiac ultrasound morphology of HFpEF model rats ($\bar{x}\pm s$).

Group	Number	Dosage (/kg)	LAVI (*B*/*A*) (mm)	LA (DI/*L*) (mm)	LA (DI/*R*) (mm)	RA (DI/*L*) (mm)	RA (DI/*R*) (mm)	IVST (mm)
WKY	10		3.20 ± 0.61	3.52 ± 0.31	3.65 ± 0.34	3.62 ± 0.40	3.61 ± 0.73	1.50 ± 0.22
SHR	9		4.02 ± 0.38^▲▲^	3.94 ± 0.95	4.26 ± 0.71^▲^	3.94 ± 0.29	3.98 ± 0.48	2.24 ± 0.30^▲▲^
HFpEF	10		4.15 ± 0.74^▲▲^	4.21 ± 0.74	4.18 ± 0.55^▲^	4.04 ± 0.51^▲^	4.08 ± 0.67	2.22 ± 0.28^▲▲^
Entresto	8	15 mg	3.27 ± 0.33^*∗*^	3.96 ± 0.53	3.65 ± 0.50	3.92 ± 0.70	3.80 ± 0.55	1.97 ± 0.33
LGQH-H	10	8.10 g	3.21 ± 0.61^*∗*^	3.46 ± 0.57	3.47 ± 0.56^*∗*^	3.58 ± 0.55	3.17 ± 0.55^*∗∗*^	2.01 ± 0.35
LGQH-L	9	4.05 g	3.32 ± 0.30^*∗*^	3.55 ± 0.29	3.59 ± 0.75^*∗*^	3.84 ± 0.73	3.54 ± 0.78	1.95 ± 0.40

(^▲^: Compared with the WKY group, *P* < 0.05. ^▲▲^: Compared with the WKY group, *P* < 0.01. ^*∗*^: Compared with the HFpEF group, *P* < 0.05. ^*∗∗*^: Compared with the HFpEF group, *P* < 0.01).

## Data Availability

The data used to support the findings of this study are available from the corresponding author upon reasonable request.

## Abbreviations

Ang II: Angiotensin II
AT1: Angiotensin II type 1 receptor
ECM: Extracellular matrix
HFpEF: Heart failure with preserved ejection fraction
HE: Hematoxylin-eosin staining
IVST: Interventricular septal thickness
LA: Left atrial
LAD: Left atrial diameter
LGQHD: Linggui Qihua decoction
LGQH-H: LGQHD high-dose group
LGQH-L: LGQHD small-dose group
MMP-9: Matrix metalloproteinase-9
RA: Right atrial
RT-PCR: Real-time polymerase chain reaction
STZ: Streptozotocin
SHR: Spontaneously hypertensive rats
TIMP-1: Tissue inhibitor of metalloproteinase-1
TGF-*β*1: Transforming growth factor *β*1.

## Supplementary Materials

Supplementary Materials Supplementary File 1. Ethical approval of LGQHD intervention in rats with heart failure.
