# Supplementary material for: Linggui Qihua Decoction Inhibits Atrial Fibrosis by Regulating TGF-β1/Smad2/3 Signal Pathway
Source: Evid Based Complement Alternat Med. 2023 Feb 11;2023:3764316. doi: 10.1155/2023/3764316 (PMC9938776; doi:10.1155/2023/3764316)
Supplement: Supplementary Materials — Supplementary File 1. Ethical approval of LGQHD intervention in rats with heart failure. [file 3764316.f1.pdf]

Medical ethics committee Approval Letter of Xiyuan Hospital, China Academy of Chinese Medical Sciences

Approval Number: Medical ethics committee of Xiyuan Hospital,China Academy of Chinese Medical Sciences,2021XLC008-3

Protocol Title	The signal transduction of heart failure with preserved ejection fraction based on the AngII/TGF- β1/Smads pathway and the intervention mechanism of traditional Chinese medicine compound		
Fund Source	National Natural Science Foundation of China(General Program)		
Research Institute	Xiyuan Hospital, China Academy of Chinese Medical Sciences		
Principal Investigator	Guoju Dong Jiangang Liu		
Review Type	Re-review	Method of Review	Expedited review
Date of Review	April 1,2021	Review Location	Xiyuan Hospital.China Academy of Chinese Medical Sciences
Approved document	1.Protocol, Version:XYYY-V-3.0,Date:30/3/2021		
Review documents	1.Re-review application<br>2.Protocol, Version:XYYY-V-3.0,Date:30/3/2021		
Validate Date	April 1,2021～March 31,2022		

Review Comment

According to the guidance on ethical treatment of experimental animals: Ministry of Science and Technology(2006)no. 398, the national experimental animal management issued by the state scientific and technological commission of the People's Republic of China in 2013,and the GB/T 35892-2018 Laboratory animal-Guideline for ethical review of animal welfare, following review of our ethics committee, we approved the ethics application.

To protect the welfare of experimental animals, Please follow the principle of replacement, reduction and refinement for treatment of experimental animals, and follow the protocol approved by the Ethics Committee.

The applicant should submit the amendment application to get re-approval of commitee ahead of time. If the principal investigator is changed during the research or any modification made with the protocol. The applicants must submit the related Serious Adverse Event report in time while there is SAE.

The applicant should submit the progress report in the month ahead of the deadline in accordance with the annual/periodic review frequency specified by the ethics committee. The applicant should submit a written report to the ethics committee in time in the event of any situation that may significantly affect experiment or increase suffering of the experimental animals.

If violation or deviation of the Protocol is occur, the applicant should submit the related reports.

Please submit the suspension/termination reports in time if the applicant intend to suspend or terminate the study in advance.

When the study is completed, the applicant is requested to submit a completion report and a summary report including the findings and conclusions of the study.

Annual/periodic review frequency	Please submit the progress report in the month before
Ethics committee	Xiyuan Hospital, China Academy of Chinese Medical Sciences
Contact telephone number	010-62835646<br>010-62835637
Signature of Committee Chair	刘建
Date	April 1,2021
